# Benchmark datasets of immune receptor-epitope structural complexes

**DOI:** 10.1186/s12859-019-3109-6

**Published:** 2019-10-10

**Authors:** Swapnil Mahajan, Zhen Yan, Martin Closter Jespersen, Kamilla Kjærgaard Jensen, Paolo Marcatili, Morten Nielsen, Alessandro Sette, Bjoern Peters

**Affiliations:** 1 Division of Vaccine Discovery, La Jolla Institute for Immunology, La Jolla, CA 92037 USA; 2 Department of Health Technology, Technical University of Denmark, Kgs. Lyngby, Denmark; 3 Instituto de Investigaciones Biotecnológicas, Universidad Nacional de San Martín, Buenos Aires, Argentina; 4 Department of Medicine, University of California, San Diego, CA 92093 USA

**Keywords:** IEDB, Epitope, Antibody, TCR, MHC, Protein structures, Epitope prediction

## Abstract

**Background:**

The development of accurate epitope prediction tools is important in facilitating disease diagnostics, treatment and vaccine development. The advent of new approaches making use of antibody and TCR sequence information to predict receptor-specific epitopes have the potential to transform the epitope prediction field. Development and validation of these new generation of epitope prediction methods would benefit from regularly updated high-quality receptor-antigen complex datasets.

**Results:**

To address the need for high-quality datasets to benchmark performance of these new generation of receptor-specific epitope prediction tools, a webserver called SCEptRe (Structural Complexes of Epitope-Receptor) was created. SCEptRe extracts weekly updated 3D complexes of antibody-antigen, TCR-pMHC and MHC-ligand from the Immune Epitope Database and clusters them based on antigen, receptor and epitope features to generate benchmark datasets. SCEptRe also provides annotated information such as CDR sequences and VDJ genes on the receptors. Users can generate custom datasets based by selecting thresholds for structural quality and clustering parameters (e.g. resolution, R-free factor, antigen or epitope sequence identity) based on their need.

**Conclusions:**

SCEptRe provides weekly updated, user-customized comprehensive benchmark datasets of immune receptor-epitope structural complexes. These datasets can be used to develop and benchmark performance of receptor-specific epitope prediction tools in the future. SCEptRe is freely accessible at http://tools.iedb.org/sceptre.

## Background

B and T cell responses are essential components of adaptive immunity which can provide long term protection against various pathogens. Antibodies and T-cell receptors (TCRs) are expressed by B and T cells, respectively, to recognize an ever-changing collection of antigens. Antibodies and TCRs recognize a specific region of the antigen, known as an epitope, with their binding site, known as a paratope. Identification of epitopes is of high importance for many medical, immunological and biological applications including disease control, diagnostics, and vaccine development [1, 2].

The best performing antigen sequence and structure-basedB-cell epitope prediction tools, such as BepiPred, DiscoTope, ABCpred and CBtope [3–6], have limited predictive power [4]. These B cell epitope prediction tools predict the surface patch on the antigen that can be a target of one or more antibodies out of likely several billion antibodies from the host. Given this immense antibody repertoire, most antigen surface patches can be targets of host antibodies and this property is one of the main reasons behind the poor performance of B cell epitope prediction methods [7]. In contrast, T cell epitope predictions rely predominantly on MHC binding predictions [8–10]. MHC binding is necessary but not enough to induce an immune response. An appropriate T-cell clone that can recognize a specific peptide-MHC (pMHC) complex is needed to induce an immune response.

Recent advances in the sequencing of immune receptor repertoire [11] have raised interest to identify epitopes by predefined antibodies and TCRs. Therefore, a new generation of B and T cell epitope prediction methods have shifted focus from predicting general epitopes in antigens to predicting epitopes for a specific receptor [12, 13]. Recently, several antibody- and TCR-specific epitope prediction methods have become available [7, 13–15]. Currently, the data needed to train and validate these methods are scarce and often repetitive. The receptor-specific epitope prediction methods utilize different clustering approaches to remove the redundancy in their training and testing datasets which makes it difficult to reliably compare and evaluate results from multiple prediction methods. There is also a need to evaluate the performance of these methods on independent datasets. However, the definition of such independent datasets is often non-trivial, because the methodologies and/or datasets used to train and develop the different tools are not completely available.

The Immune Epitope Database (IEDB) is a free public resource which captures experimental immune epitope and epitope-specific receptor data that is manually curated [16, 17]. While other 3D structural databases containing antibody and TCR information exist [18–20], the IEDB integrates these data with all other kinds of epitope mapping experiments and includes standardized definitions for the epitopes identified for each of them, includes manual quality checks for each data element, and allows users to bulk download the database. As of June 2019, IEDB has over 585,000 epitopes from over 20,300 manually curated references. IEDB also provides calculated intermolecular contacts and interface areas for 3D structures of receptor-antigen complexes. Such atomic-level details of receptor-antigen complexes are important to our understanding of the epitope recognition mechanism by immune receptors.

To address the need for high-quality datasets to benchmark performance of the receptor-specific epitope prediction tools, a webserver, called SCEptRe (Structural Complexes of Epitope-Receptor) was developed. Hierarchical clustering was used to develop comprehensive non-redundant datasets of receptor-epitope complexes based on the antigen, epitope and receptor sequence and/or structural features.

## Results

### Receptor-antigen complexes in the IEDB

The IEDB was used to extract experiments characterizing immune receptor-antigen 3D complexes. A total 2510 Ab-Ag, 296 TCR-pMHC and 1107 MHC-ligand complexes curated in the IEDB, as of June 2019, were extracted. These complexes included 319 Ab-Ag, 115 TCR-epitope-MHC and 147 MHC-ligand complexes with non-peptidic antigens, which are further discussed at the end. For peptidic epitopes, receptor-antigen 3D complexes were further filtered based on default thresholds for resolution, antigen or linear epitope sequence length and missing residues in the CDR regions as described in the following sections.

### Ab-Ag dataset

Out of all the 2510 Ab-Ag complexes, total 2191 Ab-Ag complexes with peptidic epitopes from the IEDB were filtered to remove poor resolution structures using a default 3 Å threshold. Ab-Ag complexes with antigens with less than 50 residues and missing residues in the antibody CDR regions were also removed from the dataset. Out of the 772 Ab-Ag complexes after filtering were clustered based on their antigen sequence identity, antibody CDR sequences and epitope 3D conformational similarity. Antigen sequences were clustered independently using 70% sequence identity threshold into 338 distinct groups. All available antibodies were clustered into 479 distinct groups based on their CDR sequences and their corresponding epitopes were further clustered based on the 3D conformational similarity using PocketMatch [21] into 727 distinct antibody-epitope groups (Table 1).

**Table 1:** Immune receptor-antigen 3D complexes

Type of complex	Antibody-antigen	TCR-pMHC	MHC-ligand
Total available complexes	2510	296	1107
Complexes with peptidic ligand	2191	181	960
Complexes after filtering	772	154	861
Distinct receptor-epitope pairs	727	98	439

All the receptor-antigen complexes were filtered at 3 Å resolution, along with complexes with missing residues in the antibody or TCR CDR regions. Remaining antibody-antigen complexes with antigens with less than 50 residues were further removed from the dataset. TCR-pMHC and MHC-ligand complexes with core-epitope sequences shorter than 8 residues were removed from the final dataset

### TCR-pMHC dataset

From the dataset of 181 TCR-pMHC complexes from the IEDB with peptidic epitopes, low resolution complexes were removed using 3 Å resolution threshold. TCR-pMHC complexes with core-epitopes less than 8 residues and CDRs with missing residues were also filtered out from the dataset. A total of 154 TCR-pMHC complexes after filtering were further clustered based on their core-epitope sequence similarity, CDR and MHC G-domain sequences into 105 distinct clusters including 98 distinct TCR-epitope groups (Table 1). These clusters included 63, 79 and 33 distinct groups of T cell core-epitopes, TCRs and MHC molecules, respectively.

### MHC-peptide dataset

Similar to TCR-pMHC complexes, MHC-peptide complexes with more than 3 Å resolution and less than 8 residue core-epitopes were not considered for further clustering. Out of 960 total MHC-peptide complexes, the remaining 861 MHC-peptide complexes after filtering were further clustered based on their core-peptide sequence similarity, and MHC G-domain sequences into 439 distinct clusters (Table 1). These clusters included 342 and 133 distinct groups of core-peptides and MHC molecules, respectively.

### Non-peptidic epitope or ligand datasets

A total of 319 Ab-Ag complexes with non-peptidic epitopes were filtered based on resolution and missing residues in the antibody CDR regions. The remaining antibody sequences from 281 Ab-Ag complexes were clustered into 154 distinct antibody groups based on their CDR sequences. Similarly, all the 115 TCR-pMHC complexes with non-peptidic ligands were filtered based on 3 Å resolution threshold and no missing residues in the CDR regions. The remaining 85 TCR-pMHC complexes were clustered into 28 TCR and 7 MHC distinct groups. A total of 147 MHC-ligand complexes with non-peptidic ligands were filtered based on their resolution to 126 complexes. These 126 MHC-ligand complexes were clustered based on their MHC sequences into 18 distinct groups.

### Webserver

**Fig. 1:**
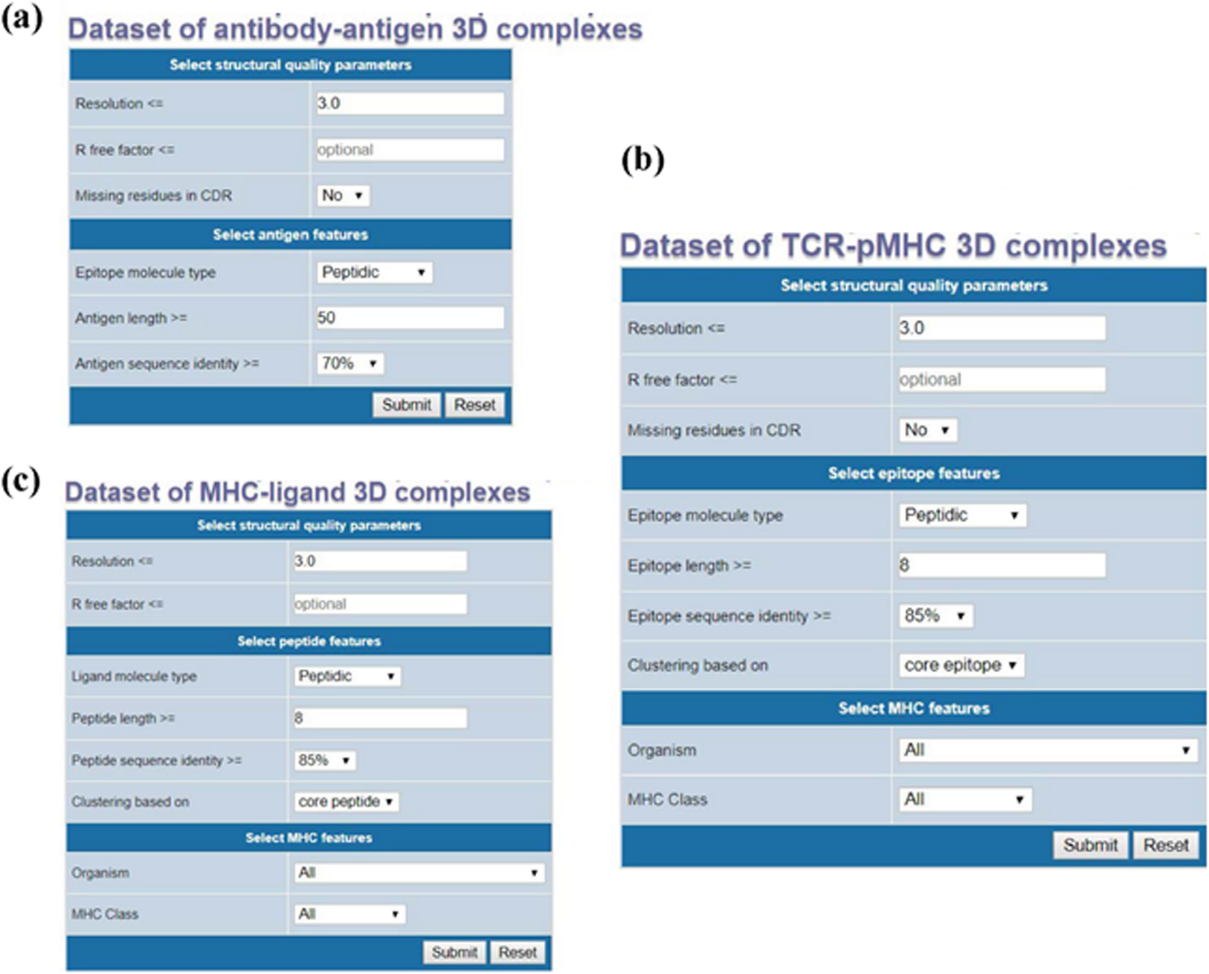
SCEptRe webserver: Users can customize various parameters to filter and cluster (**a**) antibody-antigen 3D complexes (**b**) TCR-pMHC complexes and (**c**) MHC-ligand complexes using the online resource. Recommended parameters are provided as default values in the webserver

A webserver, named SCEptRe (Structural Complexes of Epitope-Receptor), was developed and made available in the IEDB analysis resource [22] to provide weekly updated receptor-antigen 3D complexes (Fig. 1). SCEptRe can be accessed at http://tools.iedb.org/sceptre. All the structural quality parameters such as resolution, missing residues in CDRs used for filtering receptor-antigen complexes, were made available through SCEptRe. In addition, filtering based on R free factor was added to the webserver. The antigen and epitope/ligand filtering and clustering parameters were also made available. Additionally, MHC feature parameters, namely, source organism and MHC class for TCR-pMHC and MHC-ligand 3D complexes were also provided to users. An option to select receptor-antigen complexes with peptidic or non-peptidic epitopes/ligands was also added to the webserver. All the default filtering and clustering parameters discussed in the Methods sections were provided as recommended values in the online webserver, where they can be modified by the user if needed.

**Fig. 2:**
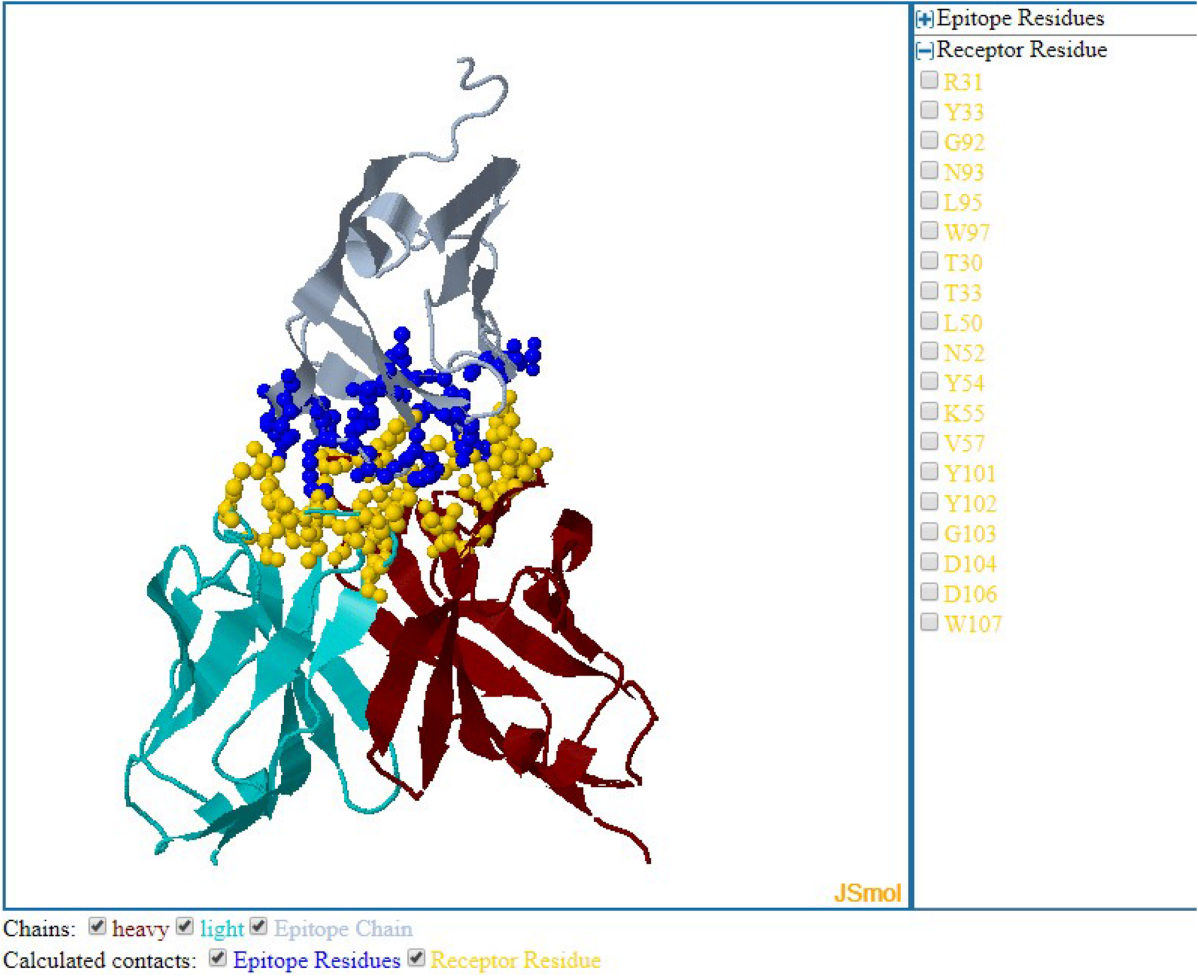
The IEDB JSmol viewer can be used to manually inspect receptor-antigen interactions. The blue colored spheres in the antibody-antigen complex (PDB ID: 1XIW) shown in the figure represent the epitope residues and yellow spheres are paratope residues in direct contact with each other. Individual interacting residues can be selected using the right-side panel

Results are shown as a table with links to IEDB assay, epitope and receptor details pages, along with a link to PDB website. The 3D structure of receptor-antigen complexes can be visualized using the IEDB JSmol viewer (JSmol: an open-source HTML5 viewer for chemical structures in 3D. http://wiki.jmol.org/index.php/JSmol) (Fig. 2) which provides options to inspect one or all interacting residues in the receptor-antigen complexes.

## Discussion

The advances in the technology to sequence B and T cell receptor repertoires from individuals has led to the development of receptor-specific B and T cell epitope prediction methods. These new epitope prediction methods would benefit from regularly updated high-qualityreceptor-antigen datasets. Resources like the IEDB provide manually curated data on experimental information of antigen-receptor complexes. SCEptRE provides several down-stream processing steps to filter data from the IEDB to retain high-qualityreceptor-antigen complexes. SCEptRE also provides approaches to clustering the receptor-antigen 3D complexes so that these datasets can be used directly to train and validate receptor-specific epitope prediction methods.

Currently available 3D receptor-antigen complex datasets cluster antibody and TCR sequences based on their full-length sequences [7, 13, 23, 24]. Antibody and TCR sequences with identical CDRs may be grouped in different clusters by such an approach. CDRs interact directly with the antigen and are linked to the specificity of B and T cell receptors [25, 26]. It is also known that antibody heavy- and TCR β chains make the most extensive contacts with the antigen. Hence, SCEptRe classifies receptors based on their CDR sequences. Receptor cluster identifiers include information on the individual heavy, light, α or β chain clusters so that users can re-cluster them based on their need, e.g. heavy chain only or β chain only groups.

SCEptRe also addresses the lack of grouping of B cell epitopes based on their 3D conformational similarity in the currently available antibody-antigen complex datasets. Cross-reactive antibody bound to conformationally similar epitopes from homologous antigens can provide similar Ab-Ag contacts residue pairs. Such Ab-Ag complexes can be grouped together by the conformational similarity between their epitopes.

Over the year, different MHC allele nomenclatures have been used by the scientific community to describe MHC molecules. SCEptRe introduces a sequence-based approach to apply a consistent classification of MHC molecules for TCR-pMHC and MHC-ligand complexes instead of relying on the reported identify of MHC molecules to provide consistent MHC molecule clusters.

## Conclusions

SCEptRe provides user-customized, weekly updated, non-redundant datasets of receptor-antigen 3D complexes along with annotated receptor information. The datasets provided by SCEptRe can be used to develop and benchmark performance of receptor-specific epitope prediction tools in the future. The clustering of the receptor-antigen complexes can be used to remove redundancy from training and validation datasets for epitope prediction algorithms.

## Methods

### Datasets

An automated query was set up which extracts the antibody-antigen, TCR-pMHC and MHC-ligand assays from the IEDB SQL database for which 3D structures were available. Information related to host organisms, source antigens, epitopes, receptor’s CDR sequences and VDJ genes along with PDB ID, resolution and calculated receptor-antigen contacts (using 4 Å distance) were retrieved. The calculated receptor-antigen contacts are inspected manually in the IEDB curation process to check and remove irrelevant contacts which are not part of antigen-receptor interactions, especially in cases of engineered antibody, TCR or MHC constructs. The R free factors of the complexes were extracted from their PDBx/mmCIF files and added to the SQL query output files. Further, all the receptor-antigen 3D complexes were filtered to remove poor quality structures. Receptor-antigen complexes with peptidic antigens (which includes full-length proteins but excludes non-peptidic small molecules) were further clustered based on their receptor and antigen features to identify distinct receptor-epitope pairs. Conversely, receptor-antigen complexes with non-peptidic epitopes were clustered separately based on their receptor features.

### Antibody-antigen (Ab-Ag) complexes

The antibody chain sequences were numbered using ANARCI [27] and CDRs were identified using the IMGT numbering [28]. Antibody CDR sequences were mapped to their 3D structures. Filters to exclude low-quality structures included, resolution (default: 3 Å) and optional removal of complexes with missing backbone atoms in the CDR regions (default: remove). Ab-Ag complexes with short antigen protein sequences can further be filtered (default: exclude antigens with < 50 amino acid residues).

The remaining Ab-Ag complexes were clustered based on their antigen sequences using the Cluster tool [29] with a sequence identity threshold (default: 70%). Furthermore, all Ab-Ag complexes were clustered based on the antibody CDR sequences. Antibody heavy and light chains with identical CDR sequences were grouped independently and their clusters were denoted using ‘H’ and ‘L’ prefixes, respectively. For example, antibody cluster H1_L1, where H1 is the first heavy chain cluster and L1 is the first light chain cluster.

PocketMatch (version 2) was used to identify conformationally similar B cell epitopes [21]. PocketMatch calculates all against all-atom distance pairs in the 2 binding sites and then compares sorted lists of distances to identify the conformationally similar binding site. Two types of scores, PMax and PMin, are provided by PocketMatch. PMax score is the ratio of matched distance pairs in the 2 binding sites over the total number of distance pairs in the longer binding site. Conversely, PMin score is the ratio of matched distance pairs in 2 binding sites over a total number of distance pairs in the shorter binding site. Both PMax and PMin scores were used to cluster B cell epitopes within each antibody cluster to generate unique antibody-epitope pairs. Pairs of B cell epitopes from each antibody cluster were clustered together if their PMax or PMin score was 1.0, or PMin score was at least 0.9 and PMax score was at least 0.6.

### TCR-pMHC complexes

Like Ab-Ag complexes, CDR sequences were identified by numbering TCR chain sequences using ANARCI with IMGT numbering. TCR-pMHC complexes were filtered to exclude low-quality structures based on resolution (default: 3 Å) and presence of missing backbone atoms in the CDR regions (default: remove). Additionally, core-epitopes were defined to ignore the overhangs of the antigenic peptides that do not directly interact with MHC or TCR molecules. The core-epitope definition included all residues between the first and last residues on the antigen chain within 4 Å distance of any atom in MHC or TCR molecules. TCR-pMHC complexes with core-epitope sequences shorter than a defined number of residues (default: 8 residues) were removed from the dataset.

The remaining TCR-pMHC were clustered based on core-epitope sequences using Cluster tool with a sequence identity threshold of (default: 85%). TCR α and β chains with identical sequences were clustered. Like antibody clustering, TCRs were clustered based on their α and β chain CDRs independently and were denoted by ‘A’ and ‘B’ prefixes, respectively.

The α1-α2 and α1-β1 domains in the MHC class I and II molecules, respectively, are known as groove or G-domains [30]. The ligand-binding site of MHC molecules are part of the G-domains and hence are responsible for their specificity. All the available G-domain sequences from IMGT [31] were downloaded. MHC chain sequences from TCR-pMHC complexes were searched against these datasets of G-domains using BLAST [32]. The BLAST parameters such as e-value of 1.00E-10, query coverage threshold of 90% and subject coverage threshold of 85% were used to align input MHC sequences to known G-domains. Regions of the MHC sequences mapped to the first G-domain hits were extracted and used to cluster TCR-pMHC complexes. MHC class I molecules with identical α1-α2 domains were clustered together and were denoted using prefix ‘a’. Similarly, MHC class II molecules with identical α1 and β1 domains were clustered together and denoted with ‘a’ and ‘b’ prefixes, respectively. For non-classical MHC molecules (for example, human CD1a), their complete protein sequences were used for clustering and the groups were denoted using ‘n’ as a prefix.

### MHC-peptide complexes

The core-peptides were defined for MHC-peptide complexes as a range of residues between the first and last residues on antigen chain within 4 Å distance of any atom in MHC molecules. MHC-peptide complexes with core-peptides shorter than 8 residues were removed from the dataset. The MHC-peptide complexes were clustered based on core-peptides using a sequence identity threshold (default: 85%), like core-epitopes in TCR-pMHC complexes. Further, these complexes were clustered based on MHC G-domains, like MHC G-domain sequence clustering in TCR-pMHC complexes.

### Complexes with non-peptidic antigens

All the complexes with non-peptidic epitopes were filtered based on resolution (default: 3 Å) and presence of missing residues in the CDR regions (default: remove). The remaining complexes were clustered based on their receptor CDR sequences and MHC molecules, where applicable, as described in earlier sections.

## Data Availability

All the data used in this paper is available at SCEptRe webserver (http://tools.iedb.org/sceptre).

## Abbreviations

Ab-Ag: Antibody-Antigen
CDR: Complementarity determining region
G-domain: Groove domain
IEDB: Immune epitope database
MHC: Major histocompatibility complex
SCEptRe: Structural Complexes of Epitope-Receptor
TCR: T cell receptor
TCR-pMHC: T cell receptor- peptide- Major histocompatibility complex

## References

[1] Fleri W, Paul S, Dhanda SK, Mahajan S, Xu X, Peters B, Sette A (2017). The immune epitope database and analysis resource in epitope discovery and synthetic vaccine design. Front Immunol.

[2] Sanchez-Trincado JL, Gomez-Perosanz M, Reche PA (2017). Fundamentals and methods for T- and B-cell epitope prediction. J Immunol Res.

[3] Jespersen MC, Peters B, Nielsen M, Marcatili P (2017). BepiPred-2.0: improving sequence-based B-cell epitope prediction using conformational epitopes. Nucleic Acids Res.

[4] Kringelum JV, Lundegaard C, Lund O, Nielsen M (2012). Reliable B cell epitope predictions: impacts of method development and improved benchmarking. PLoS Comput Biol.

[5] Saha S, Raghava GP (2006). Prediction of continuous B-cell epitopes in an antigen using recurrent neural network. Proteins.

[6] Ansari HR, Raghava GP (2010). Identification of conformational B-cell epitopes in an antigen from its primary sequence. Immunome Res.

[7] Jespersen MC, Mahajan S, Peters B, Nielsen M, Marcatili P (2019). Antibody specific B-cell epitope predictions: leveraging information from antibody-antigen protein complexes. Front Immunol.

[8] Andreatta M, Karosiene E, Rasmussen M, Stryhn A, Buus S, Nielsen M (2015). Accurate pan-specific prediction of peptide-MHC class II binding affinity with improved binding core identification. Immunogenetics.

[9] O'Donnell TJ, Rubinsteyn A, Bonsack M, Riemer AB, Laserson U, Hammerbacher J (2018). MHCflurry: open-source class I MHC binding affinity prediction. Cell Syst.

[10] Jurtz V, Paul S, Andreatta M, Marcatili P, Peters B, Nielsen M (2017). NetMHCpan-4.0: improved peptide-MHC class I interaction predictions integrating eluted ligand and peptide binding affinity data. J Immunol.

[11] Benichou J, Ben-Hamo R, Louzoun Y, Efroni S (2012). Rep-Seq: uncovering the immunological repertoire through next-generation sequencing. Immunology.

[12] Sela-Culang I, Ofran Y, Peters B (2015). Antibody specific epitope prediction-emergence of a new paradigm. Curr Opin Virol.

[13] Jurtz VI, Jessen LE, Bentzen AK, Jespersen MC, Mahajan S, Vita R, Jensen KK, Marcatili P, Hadrup SR, Peters B, et al. NetTCR: sequence-based prediction of TCR binding to peptide-MHC complexes using convolutional neural networks. bioRxiv. 2018;433706. 10.1101/433706.

[14] Sela-Culang I, Ashkenazi S, Peters B, Ofran Y (2015). PEASE: predicting B-cell epitopes utilizing antibody sequence. Bioinformatics.

[15] Zhao L, Wong L, Li J (2011). Antibody-specified B-cell epitope prediction in line with the principle of context-awareness. IEEE/ACM Trans Comput Biol Bioinform.

[16] Vita R, Mahajan S, Overton JA, Dhanda SK, Martini S, Cantrell JR, Wheeler DK, Sette A, Peters B (2019). The immune epitope database (IEDB): 2018 update. Nucleic Acids Res.

[17] Mahajan S, Vita R, Shackelford D, Lane J, Schulten V, Zarebski L, Jespersen MC, Marcatili P, Nielsen M, Sette A (2018). Epitope specific antibodies and T cell receptors in the immune epitope database. Front Immunol.

[18] Ehrenmann F, Kaas Q, Lefranc MP (2010). IMGT/3Dstructure-DB and IMGT/DomainGapAlign: a database and a tool for immunoglobulins or antibodies, T cell receptors, MHC, IgSF and MhcSF. Nucleic Acids Res.

[19] Dunbar J, Krawczyk K, Leem J, Baker T, Fuchs A, Georges G, Shi J, Deane CM (2014). SAbDab: the structural antibody database. Nucleic Acids Res.

[20] Leem J, de Oliveira SHP, Krawczyk K, Deane CM (2018). STCRDab: the structural T-cell receptor database. Nucleic Acids Res.

[21] Nagarajan D, Chandra N: PocketMatch (version 2.0): A parallel algorithm for the detection of structural similarities between protein ligand binding-sites. In: 2013 National Conference on parallel computing technologies (PARCOMPTECH). IEEE; 2013: 1–6.

[22] Dhanda SK, Mahajan S, Paul S, Yan Z, Kim H, Jespersen MC, Jurtz V, Andreatta M, Greenbaum JA, Marcatili P (2019). IEDB-AR: immune epitope database-analysis resource in 2019. Nucleic Acids Res.

[23] Ponomarenko JV, Bourne PE (2007). Antibody-protein interactions: benchmark datasets and prediction tools evaluation. BMC Struct Biol.

[24] Kringelum JV, Nielsen M, Padkjaer SB, Lund O (2013). Structural analysis of B-cell epitopes in antibody:protein complexes. Mol Immunol.

[25] Sela-Culang I, Kunik V, Ofran Y (2013). The structural basis of antibody-antigen recognition. Front Immunol.

[26] Glanville J, Huang H, Nau A, Hatton O, Wagar LE, Rubelt F, Ji X, Han A, Krams SM, Pettus C (2017). Identifying specificity groups in the T cell receptor repertoire. Nature.

[27] Dunbar J, Deane CM (2016). ANARCI: antigen receptor numbering and receptor classification. Bioinformatics.

[28] Lefranc MP, Pommie C, Ruiz M, Giudicelli V, Foulquier E, Truong L, Thouvenin-Contet V, Lefranc G (2003). IMGT unique numbering for immunoglobulin and T cell receptor variable domains and Ig superfamily V-like domains. Dev Comp Immunol.

[29] Dhanda SK, Vaughan K, Schulten V, Grifoni A, Weiskopf D, Sidney J, Peters B, Sette A (2018). Development of a novel clustering tool for linear peptide sequences. Immunology.

[30] Lefranc MP, Duprat E, Kaas Q, Tranne M, Thiriot A, Lefranc G (2005). IMGT unique numbering for MHC groove G-DOMAIN and MHC superfamily (MhcSF) G-LIKE-DOMAIN. Dev Comp Immunol.

[31] Lefranc MP, Giudicelli V, Duroux P, Jabado-Michaloud J, Folch G, Aouinti S, Carillon E, Duvergey H, Houles A, Paysan-Lafosse T (2015). IMGT(R), the international ImMunoGeneTics information system(R) 25 years on. Nucleic Acids Res.

[32] Camacho C, Coulouris G, Avagyan V, Ma N, Papadopoulos J, Bealer K, Madden TL (2009). BLAST+: architecture and applications. BMC Bioinformatics.

